# Cerebral metabolic effects of strict versus conventional glycaemic targets following severe traumatic brain injury

**DOI:** 10.1186/s13054-017-1933-5

**Published:** 2018-01-25

**Authors:** Mark P. Plummer, Natalia Notkina, Ivan Timofeev, Peter J. Hutchinson, Mark E. Finnis, Arun K. Gupta

**Affiliations:** 1 Neurosciences Critical Care Unit, Addenbrooke’s Hospital, Cambridge, CB2 0QQ UK; 2 Division of Anaesthesia, Department of Medicine, University of Cambridge, Addenbrooke’s Hospital, Cambridge, CB2 0QQ UK; 3 Division of Neurosurgery, Department of Clinical Neurosciences, University of Cambridge, Addenbrooke’s Hospital, Cambridge, CB2 0QQ UK; 4 Intensive Care Unit, Royal Adelaide Hospital, Adelaide, 5000 Australia

**Keywords:** Traumatic brain injury, Insulin, Glucose, Microdialysis

## Abstract

**Background:**

Optimal glycaemic targets for patients with severe traumatic brain injury remain unclear. The primary objective of this microdialysis study was to compare cerebral metabolism with strict versus conventional glycaemic control.

**Methods:**

We performed a prospective single-centre randomised controlled within-subject crossover study of 20 adult patients admitted to an academic neurointensive care unit with severe traumatic brain injury. Patients underwent randomised, consecutive 24-h periods of strict (4–7 mmol/L; 72–126 mg/dl) and conventional (<10 mmol/L; 180 mg/dl) glycaemic control with microdialysis measurements performed hourly. The first 12 h of each study period was designated as a ‘washout’ period, with the subsequent 12 h being the period of interest.

**Results:**

Cerebral glucose was lower during strict glycaemia than with conventional control (mean 1.05 [95% CI 0.58–1.51] mmol/L versus 1.28 [0.81–1.74] mmol/L; *P* = 0.03), as was lactate (3.07 [2.44–3.70] versus 3.56 [2.81–4.30]; *P* < 0.001). There were no significant differences in pyruvate or the lactate/pyruvate ratio between treatment phases. Strict glycaemia increased the frequency of low cerebral glucose (< 0.8 mmol/L; OR 1.91 [95% CI 1.01–3.65]; *P* < 0.05); however, there were no differences in the frequency of critically low glucose (< 0.2 mmol/L) or critically elevated lactate/pyruvate ratio between phases.

**Conclusions:**

Compared with conventional glycaemic targets, strict blood glucose control was associated with lower mean levels of cerebral glucose and an increased frequency of abnormally low glucose levels. These data support conventional glycaemic targets following traumatic brain injury.

**Trial registration:**

ISRCTN, ISRCTN19146279. Retrospectively registered on 2 May 2014.

**Electronic supplementary material:**

The online version of this article (10.1186/s13054-017-1933-5) contains supplementary material, which is available to authorized users.

## Background

Hyperglycaemia occurs frequently following traumatic brain injury (TBI) and is associated with poor outcomes [1–3]. While it is well established that marked acute hyperglycaemia drives secondary brain injury following the initial traumatic insult [4], the magnitude of the elevation in blood glucose required to cause harm remains uncertain. Current management of hyperglycaemia in TBI involves the use of intravenous infusions of short-acting insulin, titrated to maintain systemic blood glucose within target ranges that have largely been derived from randomised controlled trials in general medical, surgical or heterogeneous critically ill populations [5].

The appropriateness of applying these targets to an exclusively TBI population has been questioned as the injured brain has a heightened susceptibility to both hyper and hypoglycaemia [6, 7]. Furthermore, a small number of studies examining surrogate outcomes of cerebral glucose metabolism have suggested that intensive glycaemic control is associated with deleterious effects in patients with severe TBI, specifically an increased prevalence of brain energy crisis [8–10]. However, at present there is insufficient evidence to guide clinical practice, highlighted by the lack of a recommended glucose target in the latest iteration of the Brain Trauma Foundation’s Guidelines of the Management of Severe Traumatic Brain Injury [11].

Using cerebral microdialysis, the primary objective of this prospective randomised controlled within-subject crossover study was to compare cerebral glucose metabolism between strict and conventional glucose control; specifically this included comparisons of cerebral glucose, lactate, pyruvate and the lactate/pyruvate (L:P) ratio. The secondary objective was to determine the frequency of pathologically abnormal brain chemistry parameters between treatment phases.

## Methods

Twenty patients aged >16 years with TBI necessitating intensive care management and intracranial pressure (ICP) monitoring were recruited from the Neurosciences Critical Care Unit at Addenbrooke’s Hospital, Cambridge, UK. Recruitment occurred over two phases with ten patients recruited between August 2007 and September 2008 and ten patients recruited between August 2010 and June 2011. Written, informed assent was obtained from the patients’ next of kin. Exclusion criteria were a past medical history of diabetes mellitus (type 1 or type 2), life threatening injury where the patient was not expected to survive >48 h and pregnancy. The study was approved by the Cambridge Local Research Ethics Committee (LREC number 06/Q0108/215).

### General management protocol

All patients were intubated, mechanically ventilated and managed according to a standardised tiered head injury protocol [12, 13]. The intensity of treatment was titrated to maintain ICP <20 mmHg and cerebral perfusion pressure (CPP) >60–70 mmHg. Therapeutic options included head elevation, analgesia, sedation, muscle relaxation, ventriculostomy, moderate hyperventilation (partial pressure of carbon dioxide in arterial blood ≥ 4.0 kPa, 30 mmHg), osmotic agents, mild to moderate hypothermia (35–33 °C), CPP augmentation with vasopressors and, at the last stage, barbiturate coma and decompressive craniectomy. CPP was calculated as the difference between mean arterial pressure (MAP) and ICP.

### Monitoring

MAP was measured invasively via an arterial line with the transducer zeroed at the level of the tragus. All patients underwent monitoring of ICP with parenchymal ICP microsensors (Codman, Raynham, USA) and cerebral extracellular chemistry with microdialysis catheters (CMA70 and CMA71; CMA Microdialysis AB, Solna, Sweden). The sensors were inserted via a triple lumen access device (Technicam, Newton Abbot, UK), placed routinely in the frontal area of the right hemisphere unless clinical circumstances dictated another position.

The CMA71 and CMA70 microdialysis catheters were perfused with central nervous system perfusion fluid at the standard rate of 0.3 μl/minute, with a sampling interval of 1 h [14]. Analysis for glucose, lactate and pyruvate was performed using bedside CMA 600 or ISCUS analysers.

ICP monitoring was performed at the bedside using ICM+ software (ICM+, University of Cambridge), which in addition to recording physiological variables (ICP, MAP and CPP) allowed online calculation of the cerebrovascular pressure reactivity index (PRx). The PRx represents a moving correlation coefficient between 30 mean values of ICP and MAP, recorded at 10 second intervals, and reflects the state of cerebral vascular autoregulation; a negative correlation implies active pressure reactivity while a positive correlation suggests autoregulatory impairment [13, 14].

### Glucose protocol

Following admission to the intensive care unit (ICU), all patients were initially treated with a blood glucose control protocol using continuous infusions of intravenous insulin to target a blood glucose level <10 mmol/L (180 mg/dl) with serial arterial blood glucose measured 1- to 2-hourly (Roche OmniS, Massachusetts, USA) depending on glucose stability. Patients were randomised via computer generated assignment to an initial 24-hr period of ‘strict normoglycaemia’, (blood glucose maintained between 4–7 mmol/L; 72–126 mg/dl) [15], or conventional glucose control (<10 mmol/L; 180 mg/dl), following which they crossed-over to the alternate glycaemic target for the second 24 h period of observation with all subjects completing both arms of the crossover (Additional file 1: Figure S1 and Additional file 2: Figure S2). The first 12 h of each study period was defined as the ‘washout’ phase with the second 12 h designated a priori as the capture period of interest. Supplemental intravenous dextrose was not administered unless patients became hypoglycaemic (glucose <4 mmol/L, 72 mg/dl).

### Data processing and statistical analysis

Data were examined for known artifacts associated with flushing of the arterial line, temporary disconnection and refilling of perfusion fluid, which were manually removed [14]. Microdialysis values outside of the analytical range of the CMA-600 or ISCUS analysers (CAN, Solna, Sweden) were deemed erroneous and excluded from analysis. Summary data are presented as number (%), mean (SD) or median [IQR] as indicated. Management phase by time effects were assessed with mixed effects linear modelling and subsequent between phase comparisons performed as population-averaged effects using general estimating equations to account for repeated measures within subject; data are presented as mean (95% confidence interval). Agreement between categories was assessed using Cohen’s kappa. An α level of 0.05 was considered statistically significant. All analyses were performed in Stata MP 14.1.

The 2014 International Microdialysis Forum brain chemistry thresholds were used to define abnormal brain chemistry values; glucose <0.2 and 0.8 mmol/L, lactate >4 mmol/L and L:P ratio >25 and >40 [16]. Two separate binary logistic models were used at the pre-defined glucose microdialysis cut-points of <0.8 and <0.2.

## Results

Twenty patients were enrolled in the study with clinical details as outlined in Table 1. The cohort included 15 males, the median age was 34 [23, 50] years, Acute Physiology and Chronic Health Evaluation II score 20.5 [14.5, 23.0], injury severity score 29 [23, 40] and initial Glasgow Coma Scale score of 5 [3, 8]. ICU length of stay was 23 [17, 18] days. Two patients died on days 5 and 8, respectively. The interval between injury and initiation of microdialysis monitoring was 4.5 [3, 8] days and monitoring was continued for 4 [3, 9] days. Review of computed tomographic scans revealed that the microdialysis tip was sited in normal-appearing parenchyma in 19 patients and in peri-lesional tissue in one patient.

**Table 1:** Clinical profile of study group

Patient	Age (years)	Sex	GCS score	APACHE II score	ISS	CT scan result	ICU days	GOS^a^
1	16	Male	3	27	41	Evacuated ML	27	SD
2	32	Male	5	16	48	Diffuse	50	–
3	24	Male	3	23	25	Evacuated ML	25	–
4	54	Male	8	21	25	Non-evacuated ML	26	SD
5	39	Male	3	23	38	Diffuse	23	G
6	22	Female	11	15	9	Non-evacuated ML	12	MD
7	49	Female	5	22	17	Evacuated ML	25	G
8	55	Male	3	24	34	Diffuse	21	SD
9	22	Female	3	26	25	Non-evacuated ML	32	–
10	56	Female	8	13	16	Non-evacuated ML	21	G
11	18	Male	3	29	34	Diffuse	8	D
12	58	Male	3	9	45	Evacuated ML	5	D
13	18	Male	9	14	25	Diffuse	10	–
14	25	Male	4	17	29	Non-evacuated ML	40	SD
15	27	Male	8	23	29	Diffuse	30	–
16	43	Male	5	17	34	Diffuse	20	–
17	41	Male	12	8	16	Diffuse	15	MD
18	36	Female	10	8	20	Non-evacuated ML	19	G
19	50	Male	4	21	57	Diffuse	23	–
20	23	Male	3	20	57	Non-evacuated ML	44	–

*Abbreviations: APACHE II* Acute Physiology and Chronic Health Evaluation II, *GCS* Glasgow Coma Scale, *GOS* Glasgow Outcome Scale, *ICU* Intensive care unit, *ISS* Injury Severity Score

^a^ Glasgow Outcome Scale score at 6 months: *D* died, *G* good, *MD* moderate disability, *ML* mass lesion, *PVS* persistent vegetative state *SD* Severe disability

Mixed-effects linear models looking for treatment phase-by-time effects were non-significant, and plots of the mean variable profiles during the treatment periods were uniform. Between-period analysis was therefore undertaken for the population-averaged differences using generalised estimating equations, allowing for clustered measurements by patient.

### Serum glucose

**Fig. 1:**
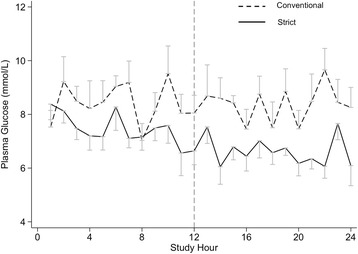
Plasma glucose time course. Commencement of the period of interest (hours 12–24) is designated by the *vertical dashed line*. Treatment separation was achieved with lower blood glucose in the strict phase (*solid line*) than in the conventional phase (*dashed line*): 6.6 (6.6–7.1) mmol/L vs. 8.4 (7.6–9.1) (*P* < 0.001). Data are presented as mean (95% CI)

**Fig. 2:**
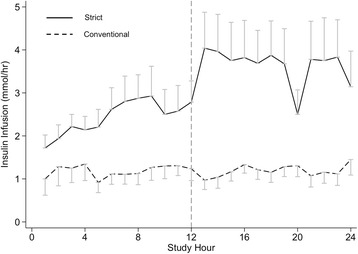
Insulin dose time course. Commencement of the period of interest (hours 12–24) is designated by the *vertical dashed line*. Strict glycaemia was associated with greater exogenous insulin infusion requirements; insulin infusion rates were 3.6 (2.2–5.0) vs. 1.1 (0.6–1.5) (*P* = 0.01). Data are presented as mean (95% CI)

In the period of interest (i.e., hours 13–24), there was clear separation in blood glucose profiles between the treatment phases (Fig. 1). The mean (95% CI) blood glucose level in the strict phase was 6.6 (6.2–7.1) mmol/L compared with 8.4 (7.6–9.1) mmol/L in the conventional phase (*P* < 0.001). There were two episodes of moderate hypoglycaemia (< 4 mmol/L) occurring in the same patient during strict glycaemia, and there were no episodes of severe hypoglycaemia (≤ 2.2 mmol/L) in either phase. Fourteen patients (70%) required exogenous insulin during the conventional phase, and 17 patients (85%) required insulin during the strict phase. The period of strict glycaemia was associated with greater exogenous insulin infusion requirements: mean (95% CI) insulin infusion rates were 3.6 (2.2–5.0) units/hr vs. 1.1 (0.6–1.5) units/hr (*P* = 0.01) (Fig. 2).

### Microdialysis results

For the strict and conventional phases, there were 10.0 [9.5–11.0] and 10.5 [9.0–11.0] time points (of a possible 12) with complete cerebral microdialysis data for glucose, lactate and pyruvate. Microdialysis glucose was lower during the strict glycaemic phase (1.05 [0.58–1.51] mmol/L versus 1.28 [0.81, 1.74] mmol/L; *P* = 0.03), as was lactate (3.07 [2.44–3.70] versus 3.56 [2.81–4.30]; *P* < 0.001). There were no significant differences in pyruvate or the lactate/pyruvate (L:P) ratio between treatment phases.

### Relationship between plasma glucose, microdialysis glucose and insulin

For a given level of arterial blood glucose, there was no association between insulin infusion rate and cerebral glucose (*P* = 0.3).

### Frequency of abnormal brain chemistry by treatment phase

**Fig. 3:**
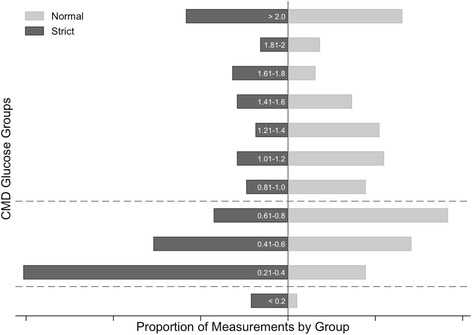
Cerebral blood glucose. Cerebral blood glucose subdivided into 0.2 mmol/L groups. Thresholds for low (< 0.8 mmol/L) and critically low (< 0.2 mmol/L) are designated by *horizontal dashed lines*. There was a trend towards increased frequency of low cerebral glucose in the 0.21–0.4 mmol/L range with strict glycaemia (OR 2.8, 95% CI 0.99–8.1; *P* = 0.05). *CMD* Cerebral microdialysis

Strict blood glucose control was associated with an increased rate of low cerebral glucose (< 0.8 mmol/L; OR 1.91, 95% CI 1.01–3.65; *P* < 0.05). There were ten episodes of critically low cerebral glucose < 0.2 mmol/L (two in the conventional arm in one patient and eight in the strict arm in six patients) (OR 4.03, 95% CI 0.69–23.40; *P* = 0.12). There were no significant differences in the frequency of abnormal L:P ratio or lactate levels between treatment phases (Table 2). A secondary analysis of the frequency of abnormal cerebral glucose was performed, dividing cerebral glucose into discrete 0.2 mmol/L subgroups (Fig. 3). Employing logistic regression, using cerebral glucose values > 2 mmol/L as the base reference group and adjusting for repeated measures, there was a trend toward an increase in the frequency of low cerebral glucose range of 0.21–0.4 mmol/L with strict control (OR = 2.80, 95% CI 0.99–8.13; *P* = 0.05) (Additional file 3: Figure S3).

**Table 2:** Measurement frequencies for International Microdialysis Forum Tier 1 substances, by treatment phase

	Group	Normal^a^	Strict^a^	Total	*P* value^b^
Glucose (mmol/L)	> 0.8	110 (6)	78 (42)	188 (50)	0.22
	0.2–0.8	79 (41)	102 (54)	181 (48)	
	< 0.2	2 (1)	8 (4)	10 (3)	
		191	188	379	
Lactate/pyruvate ratio	< 25	83 (42)	116 (60)	199 (51)	0.32
	25–40	101 (52)	67 (35)	168 (43)	
	> 40	12 (6)	9 (5)	21 (5)	
		196	192	388	
Lactate (mmol/L)	< 4	142 (73)	156 (81)	298 (77)	0.69
	≥ 4	54 (28)	36 (19)	90 (23)	
		196	192	388	

^a^ Cells are shown as number (percent) for single measurements

^b^ Probability of group effect between treatment phases by chi-square test for generalised estimating equation estimates, using a binomial distribution and logit link, clustered by patient

Summary measures for recorded variables by glucose management phase are provided in (Additional file 4: Table S1). Employing multivariate modelling by tier level, we observed that there were no associations between systemic covariates (insulin dose, partial pressure of oxygen, partial pressure of carbon dioxide, ICP, CPP, PRx, plasma glucose and plasma lactate) and markers of abnormal brain chemistry. Agreement between group levels of abnormal glucose and L:P ratio was poor (κ = 0.13), and there were no measurements with concurrent glucose and L:P ratio indicators of cerebral metabolic distress (glucose < 0.2 mmol/L and L:P ratio > 40).

## Discussion

The key finding of this study was that strict normoglycaemia, targeting a plasma glucose < 7 mmol/L (126 mg/dl), is associated with lower cerebral glucose than conventional control targeting < 10 mmol/L (180 mg/dl). There is only one previous prospective study in a TBI population providing data on brain chemistry performance according to systemic blood glucose targets [10]. In this vanguard microdialysis trial, using a within-subject crossover design, Vespa et al. [10] randomised 13 patients with severe TBI to periods of both ‘tight’ (4.4–6.1 mmol/L; 80–110 mg/dl) and ‘loose’ glycaemic control (6.7–8.3 mmol/L; 120–150 mg/dl). They reported an increase in metabolic distress during tight glycaemic control as determined by the proportion of time with critically low glucose or critically elevated L/P ratio [10]. By performing concurrent glucose-labelled positron emission tomography, they were able to demonstrate an increase in glucose metabolism during tight glycaemic control, most prominently in normal brain tissue [10]. Hence, loose glycaemic control was interpreted as being preferable for cerebral metabolism. These data are consistent with retrospective observational studies demonstrating associations between tight glycaemic control and microdialysis markers of cerebral metabolic distress [8, 9].

Analysing cerebral glucose tier levels as simple binary cut-points, strict glycaemic control increased the frequency of low cerebral glucose (cerebral glucose < 0.8 mmol/L) but not critically low cerebral glucose (< 0.2 mmol/L), albeit that there were only ten events in the critically low range. Similarly, by analysing blood glucose in discrete 0.2 mmol/L subgroups, we demonstrated a trend towards an increase in low blood glucose in the range of 0.21–0.4 mmol/L with strict glycaemic control; however, there was no difference between treatment arms in the subgroup of critically low glucose < 0.2 mmol/L. This may represent a function of the small sample size, given the low overall incidence of critically low glucose measurements (*n* = 10 [2.6%]), and we cannot exclude the possibility that a difference in cerebral chemistry may have been demonstrated with a larger sample. Furthermore, there were key differences in the glycaemic thresholds targeted and achieved in the present study compared with previous work in this area. In their prospective study, Vespa et al. achieved mean (SD) plasma glucose values of 6.1 (1.3) mmol/L, 110 (23) mg/dl and 7.7 (1.6) mmol/L, and 139 (29) mg/dl in the tight and ‘loose’ arms, respectively, reflecting contemporary practice at the time. In contrast, the mean (95% CI) values in the present study were 6.6 (6.1–7.1) mmol/L, 119 (111–127) mg/dl and 8.4 (7.6–9.1) mmol/L, and 151 (137–164) mg/dl. Importantly, in both studies, the mean systemic glucose during periods of strict glycaemia were towards the upper limit of the strict target range (6.1 and 6.6 mmol/L, respectively). Taken together, this suggests that there is a signal for harm even at higher thresholds of traditional tight glycaemia (i.e., a glucose level of 6.0 mmol/L; 108 mg/dl).

The clinical relevance of this distinction is highlighted by the movement away from the very tight glycaemic targets advocated in the seminal blood glucose trial by Van den Berghe et al. in 2001 [17]. This single-centre study in a surgical ICU population resulted in a paradigm shift in the approach to blood glucose management after demonstrating a marked reduction in mortality targeting blood glucose levels of 4.4–6.1 mmol/L; 80–110 mg/dl [17]. Despite the initial enthusiasm for this regimen, subsequent multi-centre randomised controlled trials not only failed to replicate the survival benefit but also reported higher incidences of severe hypoglycaemia [19–21] and an increase in mortality with tight control [22]. Systematic review of glycaemic targets in neurointensive care has demonstrated that strict glucose control conveys no mortality benefit compared with more liberal targets but may be associated with lower rates of poor neurological outcome despite significantly higher rates of hypoglycaemia [23]. Importantly, data from the general ICU population suggest that there is a strong dose-dependent relationship between hypoglycaemia and mortality [24]. Furthermore, mechanisms of neuronal cell death following short periods of hypoglycaemia are well established [25]. Consequently, although consensus guidelines are lacking, few ICUs would currently advocate targeting tight blood glucose control following severe TBI. We report lower cerebral glucose and a trend towards an increase in abnormally low cerebral glucose (0.2–0.4 mmol/L) when targeting systemic blood glucose of 4–7 mmol/L compared with conventional targets. Accordingly, we continue to advocate conventional glycaemic targets (< 10 mmol/L; 180 mg/dl) following acute TBI as per the NICE-SUGAR investigators’ study [22].

As a secondary outcome, we report a poor correlation between low brain glucose and high L:P ratio. Furthermore, we report no significant difference in the frequency of abnormally elevated L:P ratio by treatment phase. This differs from previous retrospective observational studies and randomised controlled trials in which researchers reported strong associations between low cerebral glucose and elevated L:P ratios, justifying combining the two as a composite end-point of so-called brain energy crisis [8–10, 26]. Our data question the appropriateness of this classification, particularly because the pathological mechanisms driving these two phenomena are likely to differ. Although incompletely understood, the L:P ratio is thought to reflect mitochondrial dysfunction, whereas low glucose likely results from increased substrate demand (i.e., greater cellular uptake and glucose use [7]) or impaired delivery.

Finally, there was no relationship between exogenous insulin administration and cerebral blood glucose. This is in contradistinction to brain chemistry studies in subarachnoid haemorrhage indicating that insulin administration per se decreases cerebral glucose independently of systemic glycaemia [27, 28]. Further studies of cerebral metabolic responsiveness to insulin during TBI are warranted.

There are several important limitations to this study. First, although this represents the largest prospective study in this area, it remains a small, single-centre study and accordingly is subject to bias, including within-subject differences such as concurrent treatments, position of the microdialysis catheter, timing of monitoring, and extent and severity of non-cerebral injuries. The small sample size also increases the risk of a type II error, possibly supported by the trend towards a significant difference in the low cerebral glucose range 0.21–0.4 mmol/L. Second, the upper 95% CI for the strict glycaemic phase (7.1 mmol/L) fell above the target range of < 7 mmol/L; although this is subtle, we cannot exclude that more vigilant adherence to ‘strict normoglycaemia’ would result in a difference in brain chemistry. Third, we did not perform continuous electroencephalography and cannot exclude the possibility that subclinical seizures may have influenced microdialysis results. Fourth, the mean time to commencement of microdialysis monitoring was 4.5 days. Accordingly, we may have failed to capture the metabolic derangements in the acute phase post-TBI. Temporal cerebral microdialysis studies indicate a pattern of decline in cerebral glucose and a rise in cerebral lactate in the week post-TBI, with day 3 post-injury thought to reflect the period of greatest metabolic demand [13, 18]. We cannot exclude that results may differ in the first 72 h post-TBI. Finally, because this is a microdialysis study, the results are subject to the limitations inherent in this focal monitoring technique whereby global assumptions of cerebral metabolism are derived from a limited sampling area. Heterogeneity in glucose use between peri-contusional and normal-appearing white matter is well described, and the degree to which cellular metabolism is oxygen-dependent is likely dynamic [7]. Multi-modality studies are required to characterise with greater granularity how altering glucose delivery influences neuronal metabolism, both within the brain and throughout recovery.

## Conclusions

In summary, strict normoglycaemia targeting a plasma glucose < 7 mmol/L (126 mg/dl) is associated with lower cerebral glucose than conventional control targeting < 10 mmol/L (180 mg/dl). There was an increase in low cerebral glucose (< 0.8 mmol/L) during strict glycaemia in secondary analysis. Future prospective studies employing larger sample sizes are required to further characterise cerebral metabolism according to systemic glycaemic targets.

## Additional files

Additional file 1: Figure S1. Strict blood glucose control insulin infusion regimen targeting a blood glucose level of 4–7 mmol/L: the Bath Protocol [15]. (PNG 199 kb)

Additional file 2: Figure S2. Conventional blood glucose control insulin infusion regimen targeting a blood glucose level < 10 mmol/L. (PNG 221 kb)

Additional file 3: Figure S3. Marginal probabilities of cerebral glucose with conventional vs. strict glycaemic control. Marginal probabilities of cerebral glucose divided into 0.2 mmol/L subgroups with glucose values > 2 mmol/L used as a reference. (PNG 78 kb)

Additional file 4: Table S1. Recorded variables by treatment phase. (DOCX 24 kb)

## Abbreviations

APACHE II: Acute Physiology and Chronic Health Evaluation II
CMD: Cerebral microdialysis
CNS: Central nervous system
CPP: Cerebral perfusion pressure
GCS: Glasgow Coma Scale
ICP: Intracranial pressure
ICU: Intensive care unit
L:P: Lactate/pyruvate ratio
MAP: Mean arterial pressure
PET: Positron emission tomography
PRx: Pressure reactivity index
TBI: Traumatic brain injury
