# Supplementary figures and images for: Cerebral metabolic effects of strict versus conventional glycaemic targets following severe traumatic brain injury
Source: Crit Care. 2018 Jan 25;22:16. doi: 10.1186/s13054-017-1933-5 (PMC5784688; doi:10.1186/s13054-017-1933-5)

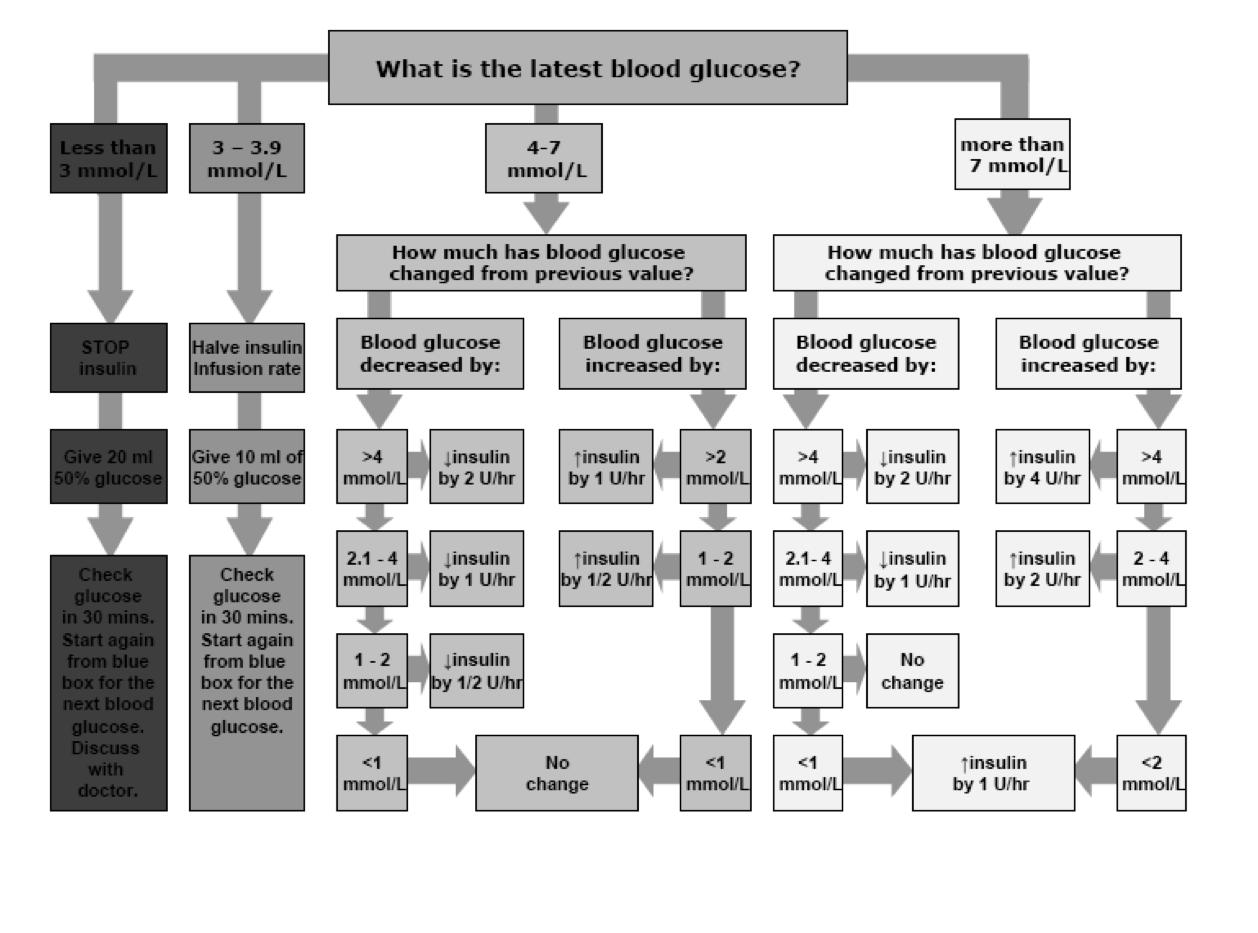

Supplement: Supplementary file 1 — Strict blood glucose control insulin infusion regimen targeting a blood glucose level of 4–7 mmol/L: the Bath Protocol [15]. (PNG 199 kb) [file 13054_2017_1933_MOESM1_ESM.png]

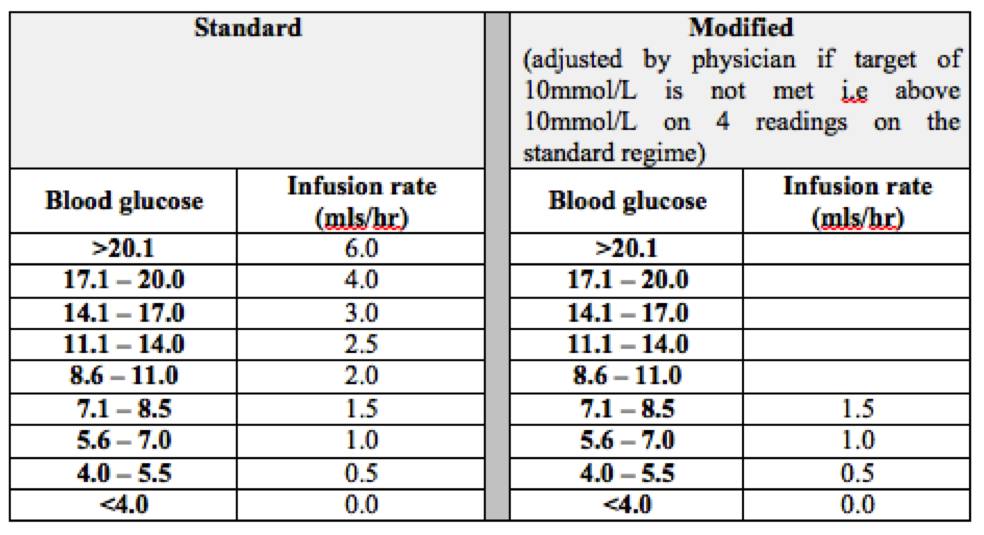

Supplement: Supplementary file 2 — Conventional blood glucose control insulin infusion regimen targeting a blood glucose level < 10 mmol/L. (PNG 221 kb) [file 13054_2017_1933_MOESM2_ESM.png]

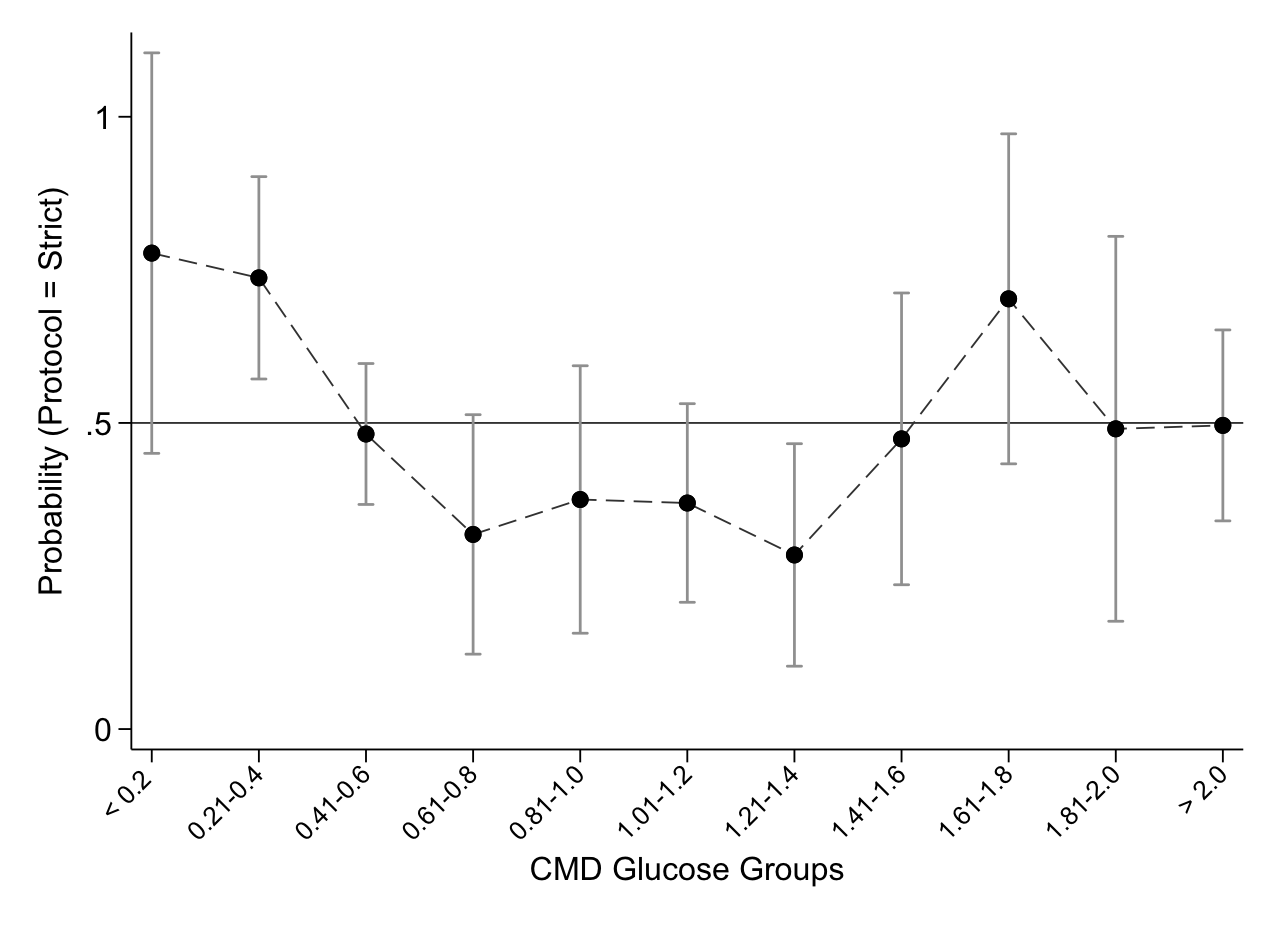

Supplement: Supplementary file 3 — Marginal probabilities of cerebral glucose with conventional vs. strict glycaemic control. Marginal probabilities of cerebral glucose divided into 0.2 mmol/L subgroups with glucose values > 2 mmol/L used as a reference. (PNG 78 kb) [file 13054_2017_1933_MOESM3_ESM.png]
