# Supplementary material for: Cerebral metabolic effects of strict versus conventional glycaemic targets following severe traumatic brain injury
Source: Crit Care. 2018 Jan 25;22:16. doi: 10.1186/s13054-017-1933-5 (PMC5784688; doi:10.1186/s13054-017-1933-5)
Supplement: Supplementary file 4 — Recorded variables by treatment phase. (DOCX 24 kb) [file 13054_2017_1933_MOESM4_ESM.docx]

| **Table S1**. Number of records and summary measures (uncorrected for within subject correlation) for recorded variables during study glucose management phases. | | | | |
| --- | --- | --- | --- | --- |
|  | **Normal Phase** | | **Strict Phase** | |
|  | **N** | **Mean (SD)** | **N** | **Mean (SD)** |
| Insulin, U/Hr | 166 | 1.2 (0.9) | 177 | 3.6 (3.1) |
| Temperature, °C | 78 | 36.5 (1.5) | 113 | 36.3 (1.4) |
| Arterial BP (Mean), mmHg | 168 | 95 (11) | 182 | 92 (11) |
| ICP, mmHg | 180 | 16 (6) | 182 | 15 (7) |
| CPP, mmHg | 168 | 79 (11) | 181 | 77 (10) |
| Pressure Reactivity Index | 128 | 0.07 (0.3) | 139 | 0.08 (0.3) |
| Arterial Blood Gas |  |  |  |  |
| PO_2_, mmHg | 82 | 105 (31) | 120 | 102 (27) |
| PCO_2_, mmHg | 82 | 37 (5) | 120 | 35 (4) |
| Glucose, mmol/L | 81 | 8.5 (2.0) | 122 | 6.6 (1.7) |
| Lactate, mmol/L | 74 | 1.2 (0.4) | 121 | 1.1 (0.4) |
| Cerebral Microdialysis |  |  |  |  |
| Glucose | 191 | 1.18 (0.89) | 188 | 1.05 (1.11) |
| Lactate | 196 | 3.4 (1.6) | 192 | 3.1 (1.5) |
| Pyruvate | 196 | 0.13 (0.04) | 193 | 0.01 (0.04) |
| L:P Ratio | 196 | 28.5 (14.2) | 192 | 26.0 (9.7) |
| Brain Tissue PO_2_ | 113 | 29.6 (10.0) | 123 | 25.9 (9.8) |
